# Liability of Physicians

**Published:** 1887-09

**Authors:** 


### LIABILITY OF PHYSICIANS.

In a recent case, involving a charge of malpractice, tried in the Supreme Court of Massachusetts, the presiding Judge in charging the jury used the following language : "Whenever men are called upon to act with dangerous agencies, the law holds them to some degree of criminal responsiblity. If they are grossly careless or reckless and presumptuous, they are guilty. The same general principle applies to medical treatment. The government must show not merely the absence of ordinary care, but gross carelessness, amounting to recklessness. A man is not to be convicted of manslaughter, merely because of his ignorance. His ignorance is only important, as bearing upon the question, whether his conduct in the care and treatment of the patient was marked by foolhardy presumptionor gross and reckless carelessness. The defendant is to be tried by no other or higher standard of skill or learning, than that which he necessarily assumed in treating her ; that is, that he was able to do so without gross recklessness or foolhardy presumption in undertaking it. It is not necessary to show an evil intent ; if by gross and reckless negligence he caused the death, he is guilty of culpable homicide." Accordingly it has been held that a dentist or surgeon using an anæsthetic, is not bound to look for any but the probable and natural effects of the drug, and is not liable for results arising from the peculiar temperament or condition of the patient, of which he had no knowledge, although if this were discoverable upon such an examination of the patient as reasonable skill and diligence require, the dentist or surgeon would be responsible for negligently failing to inform himself.

The fundamental idea on the subject is, where honesty, average intelligence, skill and learning are possessed and are applied to the treatment of the case, with ordinary diligence and caution, the physician is not liable for any mischance that may befall his patient. It is only where he has been culpable that he is liable in damages.

A physician treating a patient in good faith, to the best of his ability, is not criminally responsible for the patient's death, although caused by medicine administered by him ; but a person ignorant of the uses and properties of a poisonous drug is criminally liable for the negligent use thereof.

